# Supplementary material for: Subunit-specific NMDAR antagonism dissociates schizophrenia subtype-relevant oscillopathies associated with frontal hypofunction and hippocampal hyperfunction
Source: Sci Rep. 2018 Aug 2;8:11588. doi: 10.1038/s41598-018-29331-8 (PMC6072790; doi:10.1038/s41598-018-29331-8)
Supplement: Supplementary file 1 — Supplementary Information [file 41598_2018_29331_MOESM1_ESM.pdf]

Subunit-specific NMDAR antagonism dissociates schizophrenia subtype-relevant oscillopathies associated with frontal hypofunction and hippocampal hyperfunction.

Benjamin Pittman-Polletta[1,2,3*], **Kun Hu**[1,2], **and Bernat Kocsis**[1,4]

[1]Harvard Medical School, Boston, MA
[2]Brigham & Women's Hospital, Boston, MA
[3]Boston University, Boston, MA
[4]Beth Israel Deaconess Medical Center, Boston, MA
[*]benpolletta@gmail.com

Supplementary Information

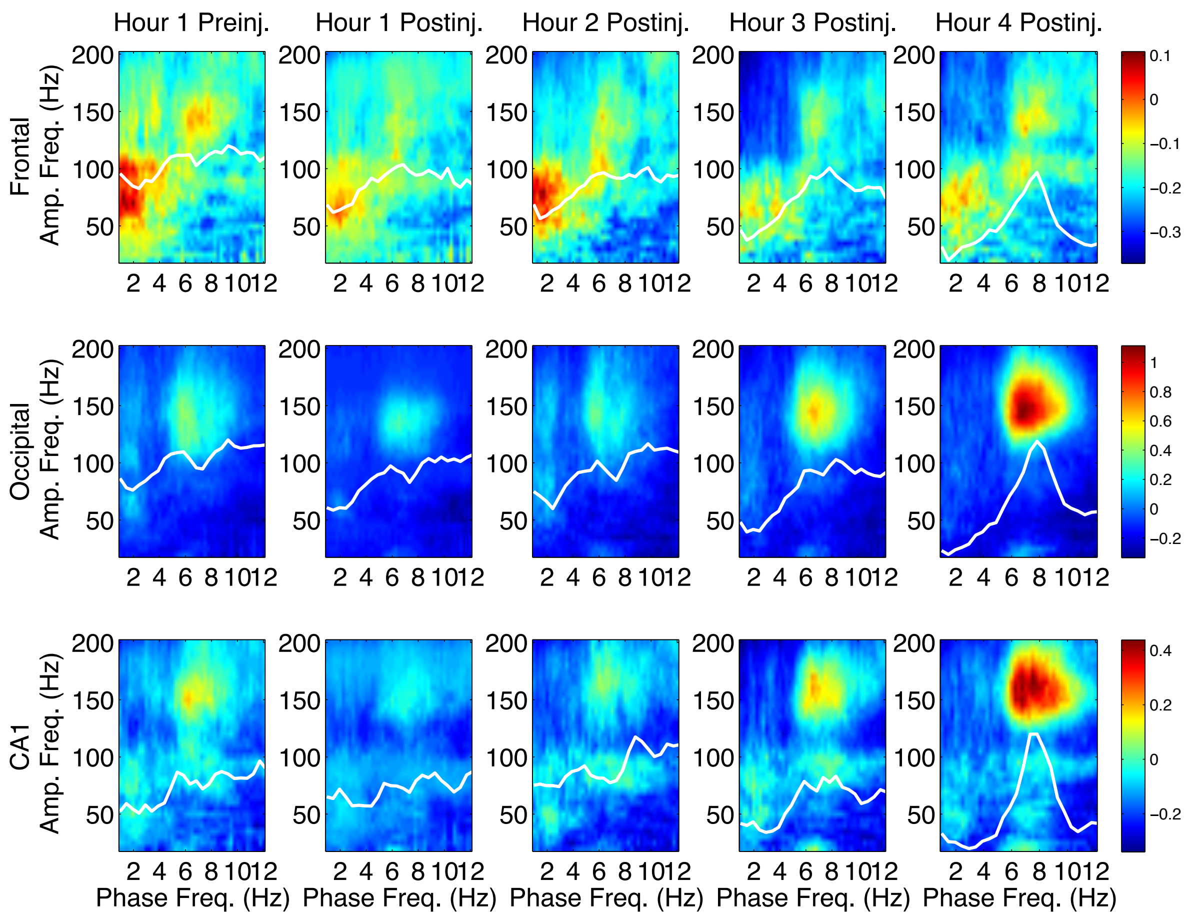

Figure S1. **Region-specific PAC patterns following saline injection.** Median (n=6) comodulograms for the 1 h preceding and the 4 h following saline injection, shown for frontal (top), occipital (middle), and CA1 (bottom) electrodes (simultaneous recordings). Overlaid white traces show the mean spectral power at frequencies below 12 Hz for each h.

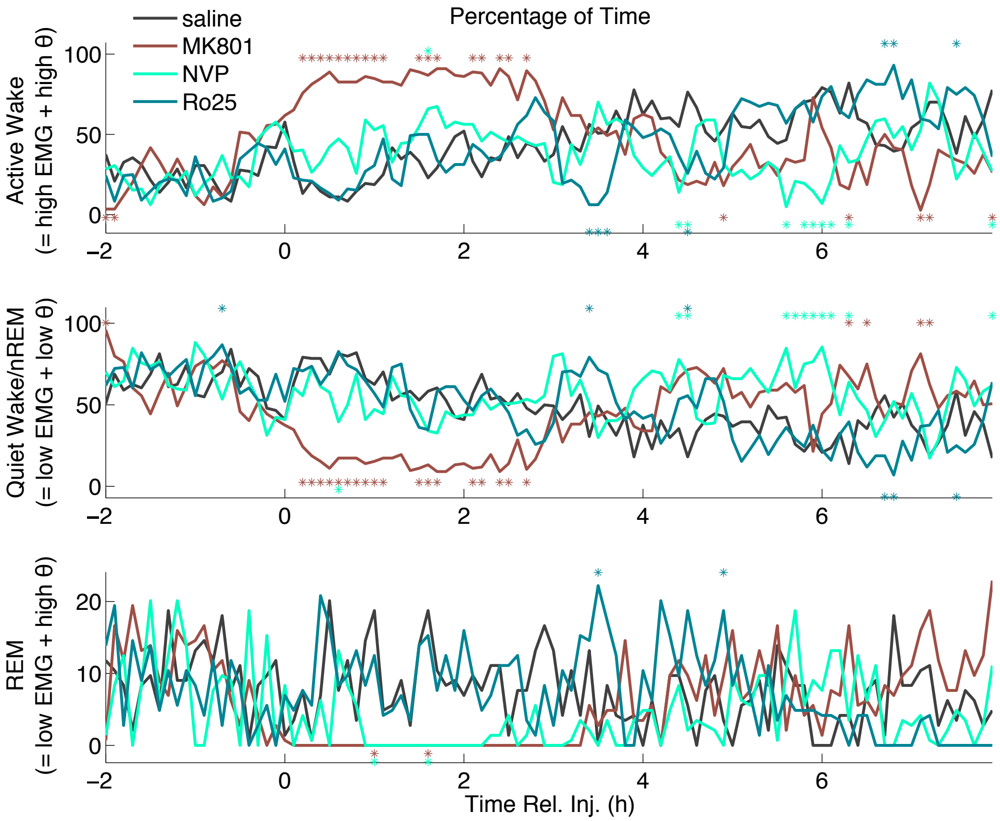

Figure S2. **Vigilance states following drug administration.** Median (n=6) percentage of each 6 min period spent in AW (defined as high EMG and θ power; top), QW/nREM sleep (defined as low EMG and θ power, and including quiet waking as well as slow-wave sleep periods; middle), and REM (defined as atonia and high θ power; bottom), following injection with saline, MK-801, NVP-AAM077, and Ro25-6985. Stars indicate 6 min periods for which spectral power is significantly higher (above) or lower (below) than saline (ranksum test, significance level = 0.05).

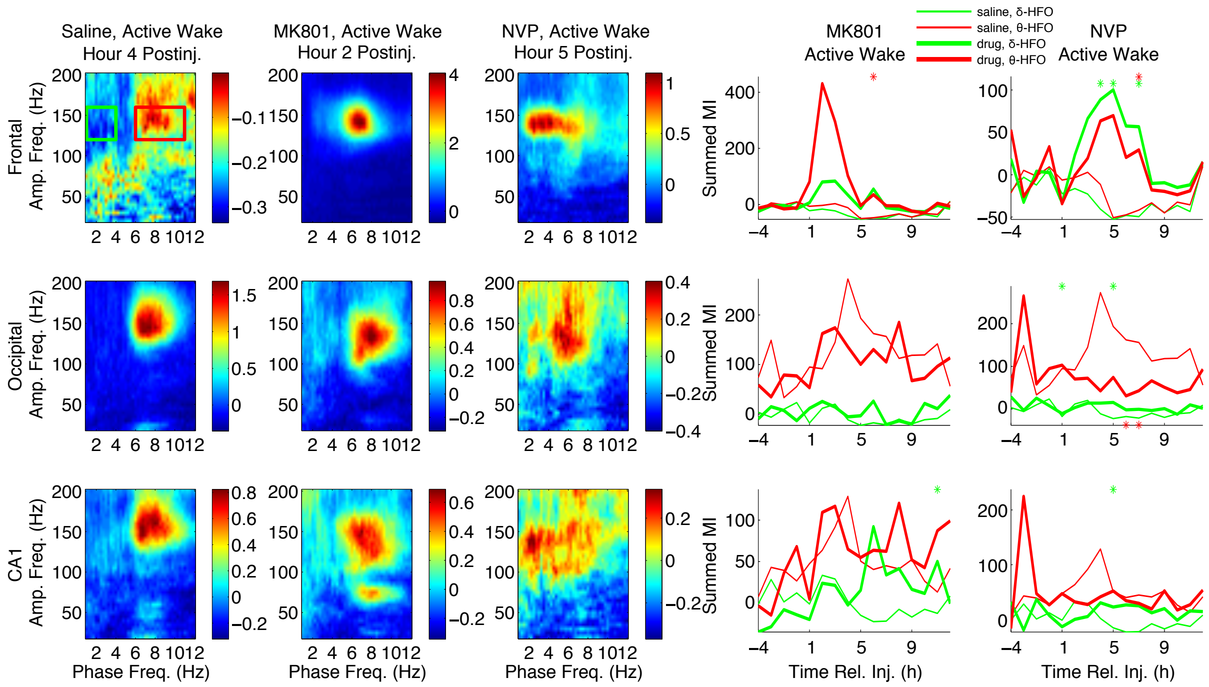

Figure S3. **Patterns of phase-amplitude coupling following MK-801 and NVP-AAM077 injection differ markedly during AW epochs.** Left three columns show median (n=6) comodulograms for all AW epochs during the h of largest effect for simultaneous LFP recordings from frontal (top), occipital (middle), and CA1 (bottom) electrodes, following injection of saline, MK-801, and NVP-AAM077. Right two columns show timeseries of summed PAC over the θ-HFO (red rectangle and time series) and δ-HFO (green rectangle and time series) frequency ranges for all AW epochs from 4 h pre-injection to 12 h post-injection. Thick lines are drug time series; thin lines are saline time series; stars indicate 6 min periods for which drug PAC is significantly higher (above) or lower (below) than saline (ranksum test, significance level = 0.05).

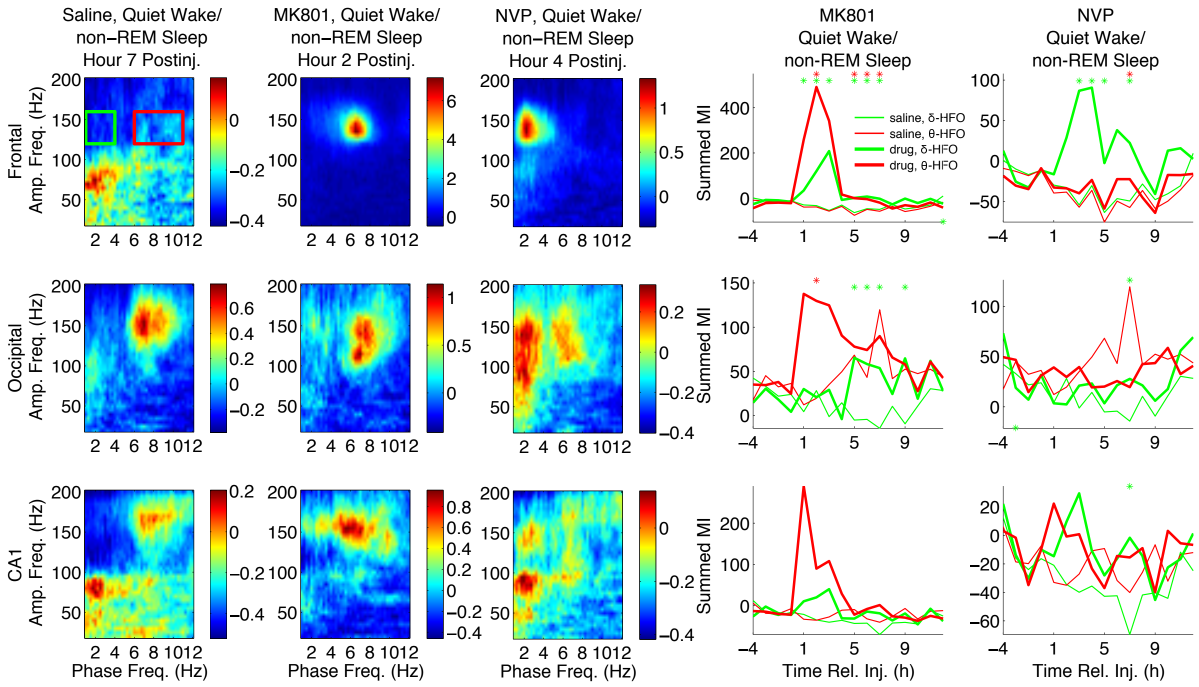

Figure S4. **Patterns of phase-amplitude coupling following MK-801 and NVP-AAM077 injection differ markedly during QW/nREM epochs.** Left three columns show median (n=6) comodulograms for all QW/nREM epochs during the h of largest effect for simultaneous LFP recordings from frontal (top), occipital (middle), and CA1 (bottom) electrodes, following injection of saline, MK-801, and NVP-AAM077. Right two columns show timeseries of summed PAC over the θ-HFO (red rectangle and time series) and δ-HFO (green rectangle and time series) frequency ranges for all QW/nREM epochs from 4 h pre-injection to 12 h post-injection. Thick lines are drug time series; thin lines are saline time series; stars indicate 6 min periods for which drug PAC is significantly higher (above) or lower (below) than saline (ranksum test, significance level = 0.05).

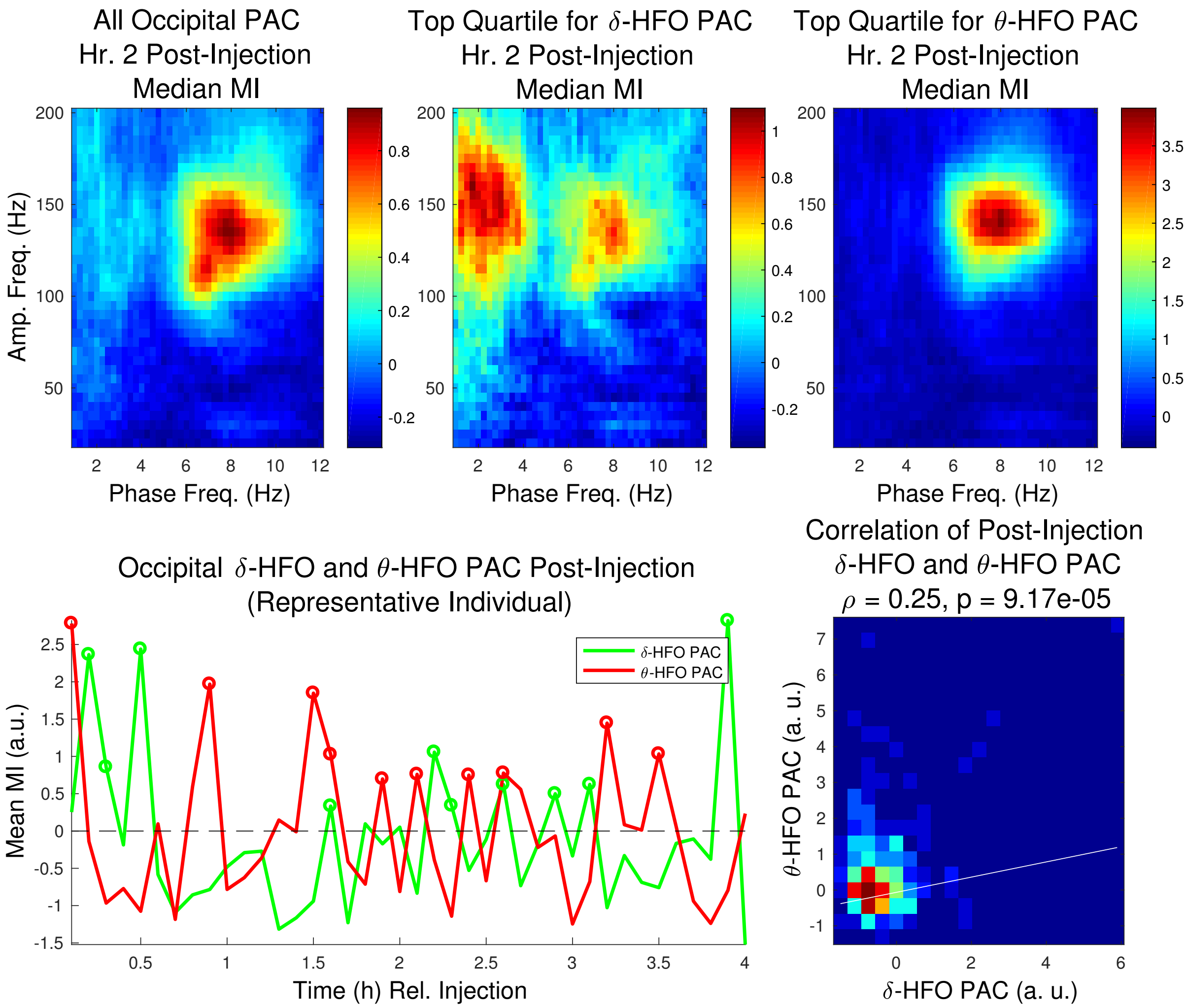

Figure S5. **δ-HFO PAC and θ-HFO PAC are correlated in occipital cortex following MK-801 administration.** Top left, median occipital comodulograms in the 2nd h following MK-801 injection. Top middle and right, median occipital comodulograms for the top quartile of epochs with respect to δ-HFO PAC and θ-HFO PAC. Bottom left, time courses of summed δ- and θ-HFO PAC for 6 min. periods following injection (representative animal, circles indicate 6 min. periods in the top quartile). Bottom right, the correlation between δ- and θ-HFO PAC over all animals.

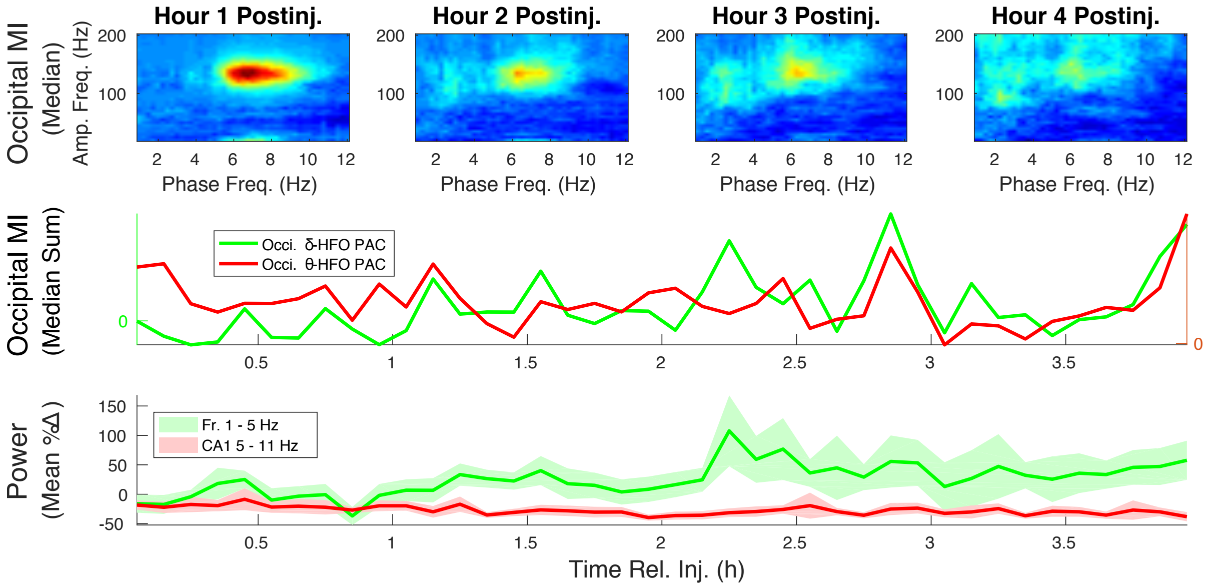

Figure S6. **The time courses of frontal δ power and CA1 θ power mirror the time courses of δ-HFO PAC and θ-HFO PAC, respectively, during AW epochs.** Top, median (n=6) occipital comodulograms are shown for AW epochs during the first 4 h following NVP-AAM077 injection. Middle, profiles of median (n=6) summed PAC during AW epochs (at 6 min intervals) are shown for δ-HFO and θ-HFO ranges. Bottom, mean $\pm$ s.d. (n=6) frontal δ power (summed over 1–5 Hz) is plotted alongside median (n=6) CA1 θ power (summed over 5–11 Hz) for AW epochs.

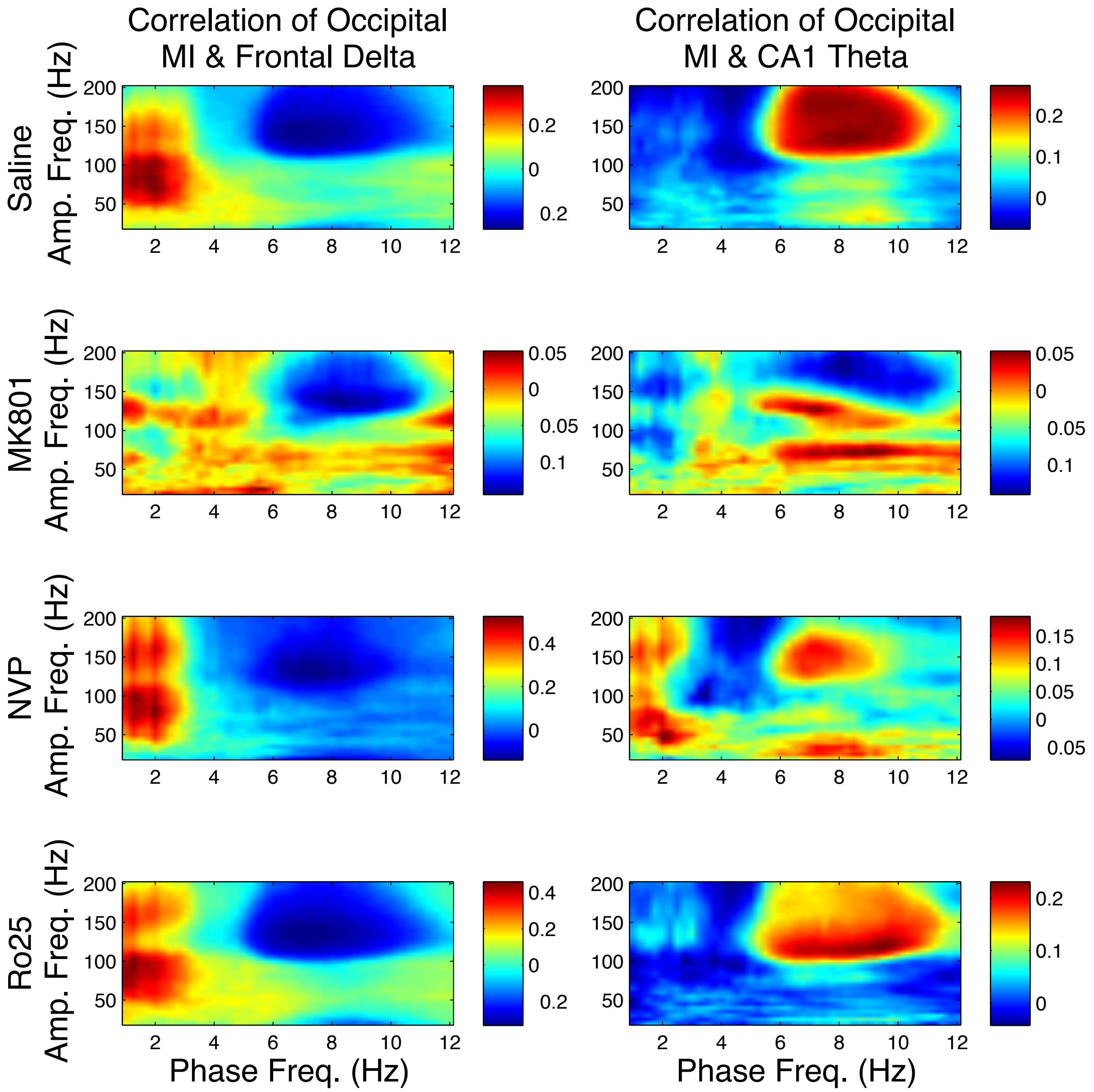

Figure S7. **Frontal δ and CA1 θ are correlated with δ-HFO and θ-HFO PAC.** The correlation between PAC MI and Frontal δ power (left) or CA1 θ power (right) during the first 4 h following injection are shown for each pair of phase-giving and amplitude-giving frequencies, and for each channel, frontal (top), occipital (middle), and CA1 (bottom).

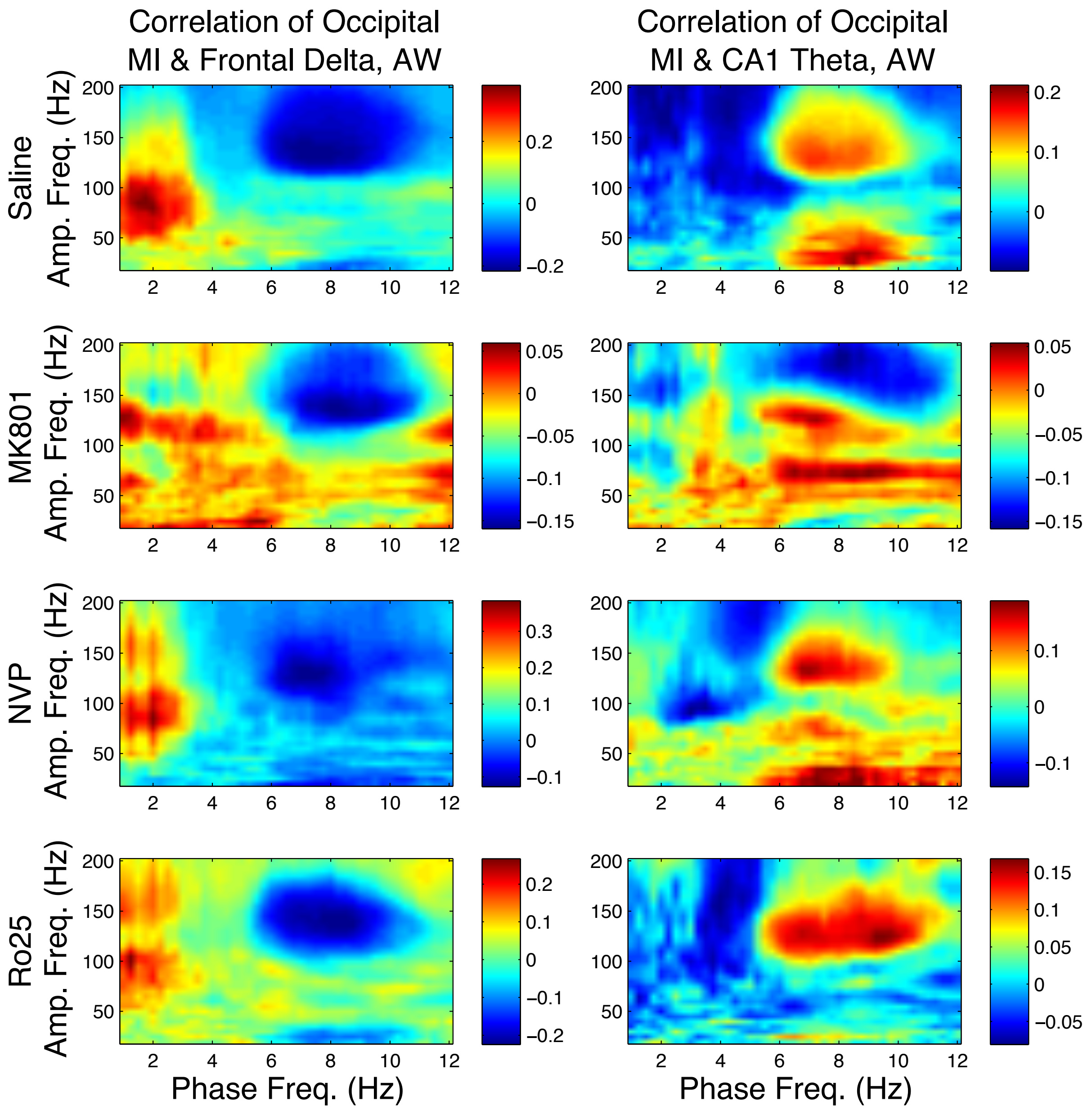

Figure S8. **Frontal δ and CA1 θ are correlated with δ-HFO and θ-HFO PAC during AW epochs.** The correlation between PAC MI and Frontal δ power (left) or CA1 θ power (right) during AW epochs for the first 4 h following injection are shown for each pair of phase-giving and amplitude-giving frequencies, and for each channel, frontal (top), occipital (middle), and CA1 (bottom).
